# Characterization of the Sialic Acid Binding Activity of Influenza A Viruses Using Soluble Variants of the H7 and H9 Hemagglutinins

**DOI:** 10.1371/journal.pone.0089529

**Published:** 2014-02-21

**Authors:** Anne-Kathrin Sauer, Chi-Hui Liang, Jürgen Stech, Ben Peeters, Pascale Quéré, Christel Schwegmann-Wessels, Chung-Yi Wu, Chi-Huey Wong, Georg Herrler

**Affiliations:** 1 Institute of Virology, University of Veterinary Medicine, Hannover, Germany; 2 Genomics Research Center, Academia Sinica, Taipei, Taiwan; 3 Friedrich-Loeffler-Institut, Institute of Molecular Biology, Greifswald Insel Riems, Germany; 4 Central Veterinary Institute, Lelystad, Netherlands; 5 Institut National de la Recherche Agronomique Centre Val de Loire, Unité mixte de recherche 1282, Infectiologie et Santé Publique, Nouzilly, France; Kantonal Hospital St. Gallen, Switzerland

## Abstract

Binding of influenza viruses to target cells is mediated by the viral surface protein hemagglutinin. To determine the presence of binding sites for influenza A viruses on cells and tissues, soluble hemagglutinins of the H7 and H9 subtype were generated by connecting the hemagglutinin ectodomain to the Fc portion of human immunoglobulin G (H7Fc and H9Fc). Both chimeric proteins bound to different cells and tissues in a sialic acid-dependent manner. Pronounced differences were observed between H7Fc and H9Fc, in the binding both to different mammalian and avian cultured cells and to cryosections of the respiratory epithelium of different virus host species (turkey, chicken and pig). Binding of the soluble hemagglutinins was similar to the binding of virus particles, but showed differences in the binding pattern when compared to two sialic acid-specific plant lectins. These findings were substantiated by a comparative glycan array analysis revealing a very narrow recognition of sialoglycoconjugates by the plant lectins that does not reflect the glycan structures preferentially recognized by H7Fc and H9Fc. Thus, soluble hemagglutinins may serve as sialic acid-specific lectins and are a more reliable indicator of the presence of binding sites for influenza virus HA than the commonly used plant lectins.

## Introduction

The importance of N-acetylneuraminic acid as a receptor determinant for influenza A viruses has been known for more than 50 years, when the viral receptor-destroying enzyme was shown to release this sugar from mucins [1]. Later it was found that influenza viruses may differ in their preference for a certain type of sialic acid, e.g. N-acetyl or N-glycolylneuraminic acid [2]. Further variation in the preferential binding activities has been attributed to the linkage type that connects the terminal neuraminic acid residue of a sialoglycoconjugate to the penultimate galactose [3]. Alpha2,6-linked sialic acids are present on oligosaccharides that are recognized by human influenza viruses [3], [4]. Most avian influenza viruses have a preference for receptors that contain the receptor determinant in an α2,3-linkage [3], [4]. However, also several avian influenza viruses of the H9 subtype, especially those isolated from land-based host animals like quails and turkeys, have been shown to recognize α2,6-linked sialic acids efficiently [5]. One or few amino acid exchanges in the viral surface protein hemagglutinin may determine which linkage type is preferentially recognized [6]. Such mutations may occur during viral adaptation to different species and thus may pave the way to successful transmission to a new host.

Despite detailed information about the receptor-binding site of the influenza hemagglutinin and about the binding preferences of different influenza viruses, the cellular receptor for these viruses is not known (for a review see [7]). Both glycoproteins and glycolipids may serve as attachment sites and initiate the entry process. For influenza A and B viruses it is not known how many surface glycoproteins/glycolipids are involved in the initiation of an infection. For influenza C virus which recognizes a less frequent type of sialic acid, N-acetyl-9-O-acetylneuraminic acid, we demonstrated that a mucin-type glycoprotein, gp36 on some human and gp40 on some canine cells, is the major surface protein recognized by this virus [8]. As this protein can also mediate endocytotic uptake, it has the characteristics of a receptor for influenza viruses [9].

In order to understand the interaction of influenza viruses with its host, it is necessary to know the distribution of sialic acids, both the type of neuraminic acid and the linkage type on the surface of the target cells. Two plant lectins, the *Maackia amurensis* agglutinin (MAA) and the *Sambucus nigra* agglutinin (SNA) are commonly used to differentiate between these linkage types. MAA recognizes α2,3-linked and SNA α2,6-linked sialic acids. Using those lectins it has been shown that avian respiratory epithelial cells primarily express the α2,3-linkage type on the cell surface [10], [11]. However, in the upper airways of pigs and humans, the predominant linkage type is α2,6, whereas further down to the lower airways, a steady increase in MAA staining indicates a substantial portion of α2,3-linked sialic acids [12]–[14]. Such studies yielded rather general information about the distribution of sialic acid linkage types. However, considering the huge variety of oligosaccharide structures, the plant lectins may not bind to all of them or with different affinities. Moreover, the sialic acids recognized by MAA and SNA may be different from those that interact with mammalian or avian influenza virus hemagglutinins, respectively. Therefore, binding studies with plant lectins may not provide correct information about the presence of receptors for influenza viruses.

To assess the binding sites for influenza viruses on the surface of target cells, we generated soluble hemagglutinins and used them for binding studies with either immortalized cells or cryosections from the avian and porcine lung. Binding of the soluble hemagglutinins was compared with that of intact virions and plant lectins. Our results demonstrate that soluble hemagglutinins are valuable tools to reveal the binding sites for influenza viruses on host cells and tissues.

## Results

### Expression and purification of soluble hemagglutinins

**Figure 1:**
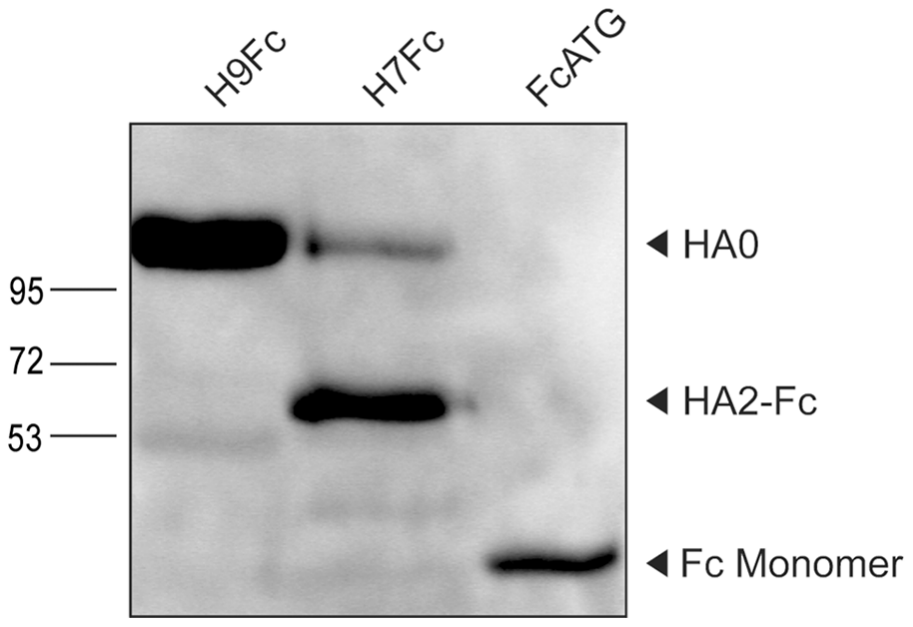
Western blot of soluble hemagglutinins H7Fc and H9Fc. Soluble hemagglutinins were prepared from the supernatant of transfected HEK 293T cells, after separation by SDS PAGE under reducing conditions. Proteins were detected using peroxidase conjugated anti-human IgG directed against the Fc-tag.

Soluble hemagglutinins (HA) were generated by connecting the ectodomains of an H7 and an H9 HA with the Fc portion of a human IgG and transfection of the cDNA constructs in HEK293T cells. After expression in the absence of trypsin, H9Fc was detected in the supernatant by SDS PAGE under reducing conditions and Western blotting with an antibody directed against Fc as a major band of about 100 kDa (Fig. 1). The H7 protein originated from a highly pathogenic influenza virus and is, therefore, cleaved intracellulary by furin-like proteases into the subunits HA1 and HA2 [23]. Under the reducing conditions of the analysis, the disulfide bonds are cleaved and H7Fc is predominantly detected as a band of about 60 kDa which corresponds to the HA2Fc fragment. An upper band of about 100 kDa represents a minor amount of uncleaved H7Fc. As the proteins were visualized by immunodetection of Fc which is connected to the carboxyterminus of HA2, the HA1 subunit cannot be detected in this blot. The Fc portion is visible as a band of about 30 kDa corresponding to monomers of the immunoglobulin heavy chain. Treatment of H9Fc with trypsin resulted in a protein of about 60 kDa corresponding to the size of HA2Fc (not shown). Taken together, by connecting the C-terminus of the H7 and H9 HA ectodomains with the Fc domain, we obtained soluble and detectable forms of the H7 and H9 HAs.

### Binding to cells

Binding tests of soluble HAs were performed with different cell types of mammalian and avian origin. MDCKII cells were chosen, because they are often used to propagate influenza viruses. Calu-3 and A549 cells are human cell lines originating from the respiratory epithelium, the primary target of influenza virus infections. CLEC213 is a cell line derived from the lung of chicken, and primary chicken kidney cells (PCKC) are epithelial cells isolated from the kidneys of chicken embryos which are commonly used for propagation of avian viruses.

**Figure 2:**
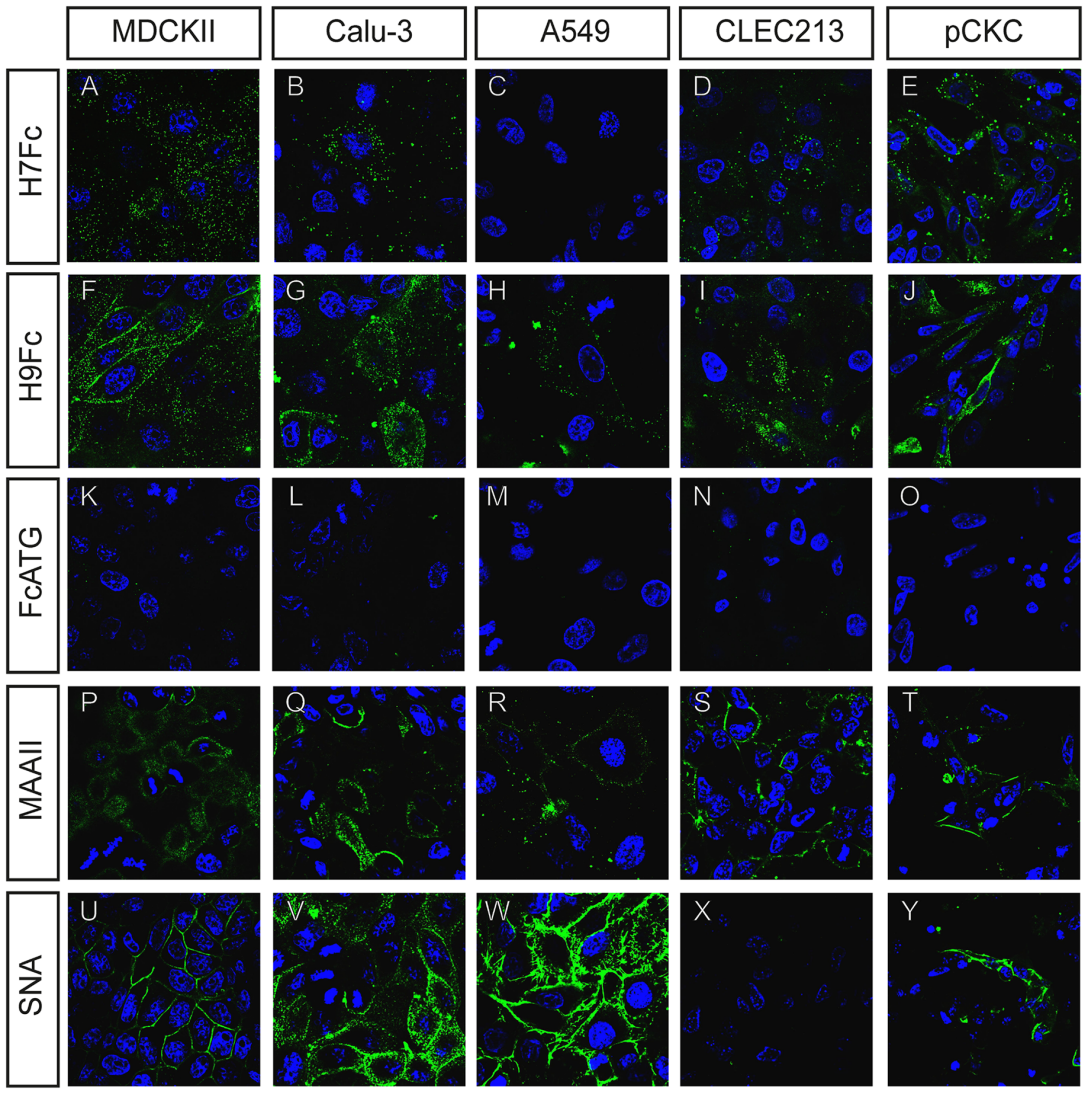
Immunofluorescence binding test of soluble HAs on mammalian and avian cells. Cells were incubated with 100 pmol of the respective soluble HA or the control FcATG. Soluble HAs were detected using anti-human-FITC antibody and nuclei were stained with DAPI. In addition, lectin staining was performed to display the presence of α2,3 (Biotin conjugated MAAII and Streptavidin-FITC) and α2,6 linked sialic acids (FITC conjugated SNA).

Lectin staining with MAAII and SNA showed that most cell lines express both α2,3 and α2,6-linked sialic acids, respectively (Fig. 2,P–Y). All five cell types analyzed showed binding of MAAII with some variations as far as the intensities of the fluorescent signals are concerned (Fig. 2P–T). Pronounced differences were observed when the binding of SNA was analyzed. With Calu-3 and A549 cells, SNA staining was stronger (Fig. 2V and 2W) than the MAAII staining, whereas MDCKII and PCKC cells show equal signal intensities with both lectins (Fig. 2 P, U, T, and Y). Remarkably, the CLEC213 cells showed MAAII staining only and no SNA staining (Fig. 2S and X). No staining was observed with the negative control FcATG (Fig. 2K–O) confirming the specificity of the observed fluorescence signals.

H9Fc bound to the surface of all cells (Fig. 2F–J), most efficiently to MDCKII, Calu-3 and CLEC213 (Fig. 2F,G,I) cells, less efficiently to A549 cells with relative fluorescence signals of about 16% when compared with the binding to MDCKII cells (Fig. 2H and data not shown). In general, binding of H7Fc was similar but less efficient with all cells, although both HAs were applied at equal molarities (overall H9Fc showed 2–3 times higher relative fluorescence on all cell lines). Weakest binding of H7Fc was observed with A459 cells with only 3% relative fluorescence compared to MDCKII cells which was hardly above background levels (Fig. 2C and data not shown). The difference between the two hemagglutinins was not restricted to the intensity of the signals but applied also to the pattern of fluorescence. Whereas H7Fc binding was characterized by large fluorescent dots distributed over the cells, staining of MDCKII and PCK cells by H9Fc was also found at the sites of cell-cell contact (Fig. 2, compare A,E and F,J). A differential fluorescence pattern was also observed when the binding of the HAs was compared to that of the plant lectins. This can be seen for example in the case of MDCKII cells where the sharp fluorescent lines corresponding to cell-cell contacts were observed after both H9Fc and SNA staining but not by MAAII staining. On the other hand, the dotted staining pattern that is also present in the H9Fc sample is lacking after staining with SNA. Overall, the soluble HAs can be utilized for probing binding sites for influenza hemagglutinins on cultured cells.

### Pre-treatment with neuraminidase

**Figure 3:**
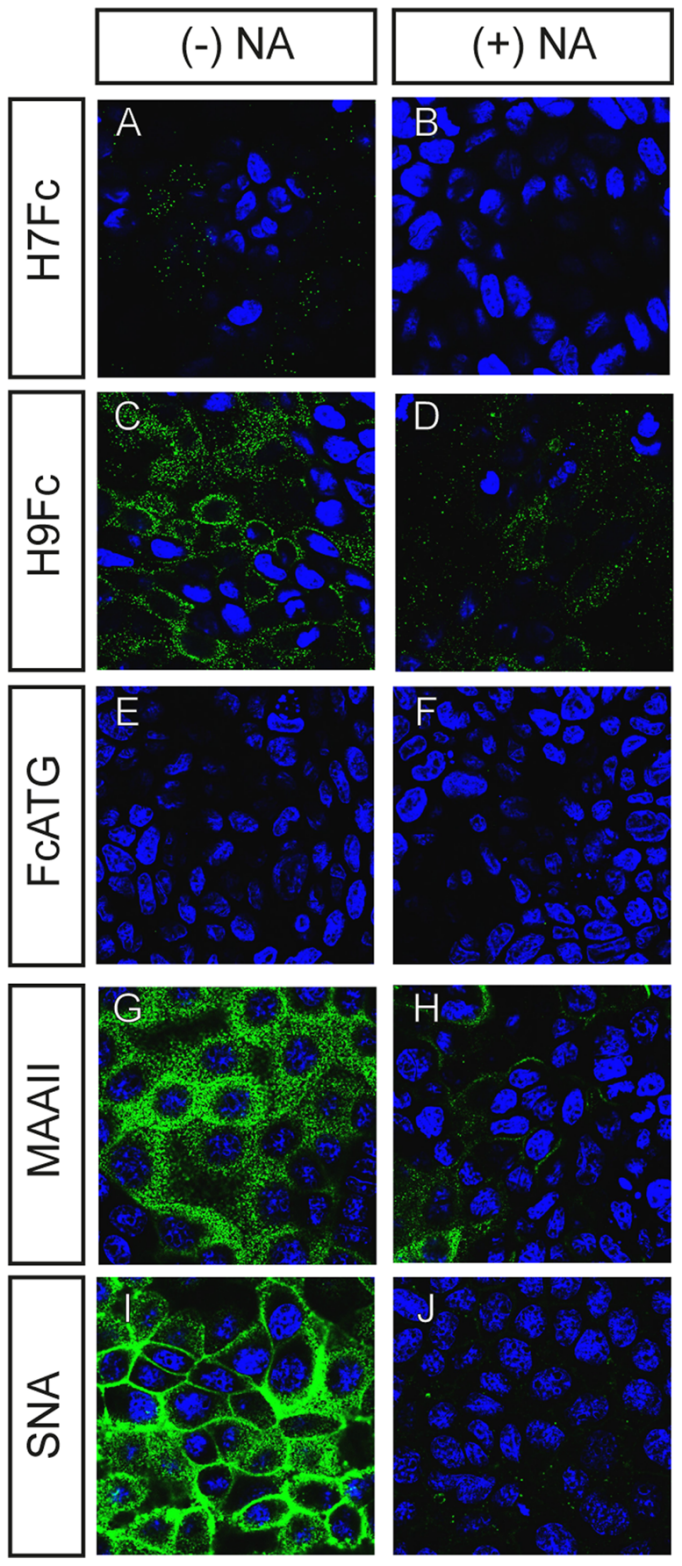
Binding to MDCKII cells after neuraminidase pretreatment. Cells were incubated with 200*Clostridium perfringens* for 1 h at 37°C. After enzyme treatment, cells were fixed and binding tests and lectin staining were performed.

To determine whether the binding of the soluble HAs is sialic acid-dependent, we pre-treated MDCKII cells with neuraminidase to remove sialic acids from the cell surface (Fig. 3). The weak H7Fc binding is abolished by prior desialylation of cells (Fig. 3A,B). H9Fc binding is clearly decreased; only some residual staining can still be observed (Fig. 3C,D). As expected, the staining with plant lectins was also sialic acid-dependent. There is a clear decrease in the binding of MAAII to neuraminidase-treated cells (Fig. 3G,H). In the case of SNA, the effect is even more pronounced (Fig. 3I,J). Taken together, similar to plant lectins, the binding of soluble HAs to cells is sialic acid-dependent.

### Binding of virus

To compare the binding of the soluble hemagglutinins with virus binding, the cell lines mentioned above were also used for binding tests with intact virions. The H9N2 virus used was the strain from which the soluble H9Fc was derived. The H7N7 virus used is a low-pathogenic H7N7 virus (A/duck/Potsdam/15/90). The strain A/chicken/Netherlands/ 621557/2003 from which H7Fc is derived is highly pathogenic and requires BSL3 conditions. Both H7 hemagglutinins showed 96% amino acid sequence identitiy in bl2seq alignment (http://blast.ncbi.nlm.nih.gov/Blast.cgi) and key amino acids within the receptor binding site were identical (data not shown). Virus was applied to cell lines at a concentration of 5×10^5^ ffu/ml.

**Figure 4:**
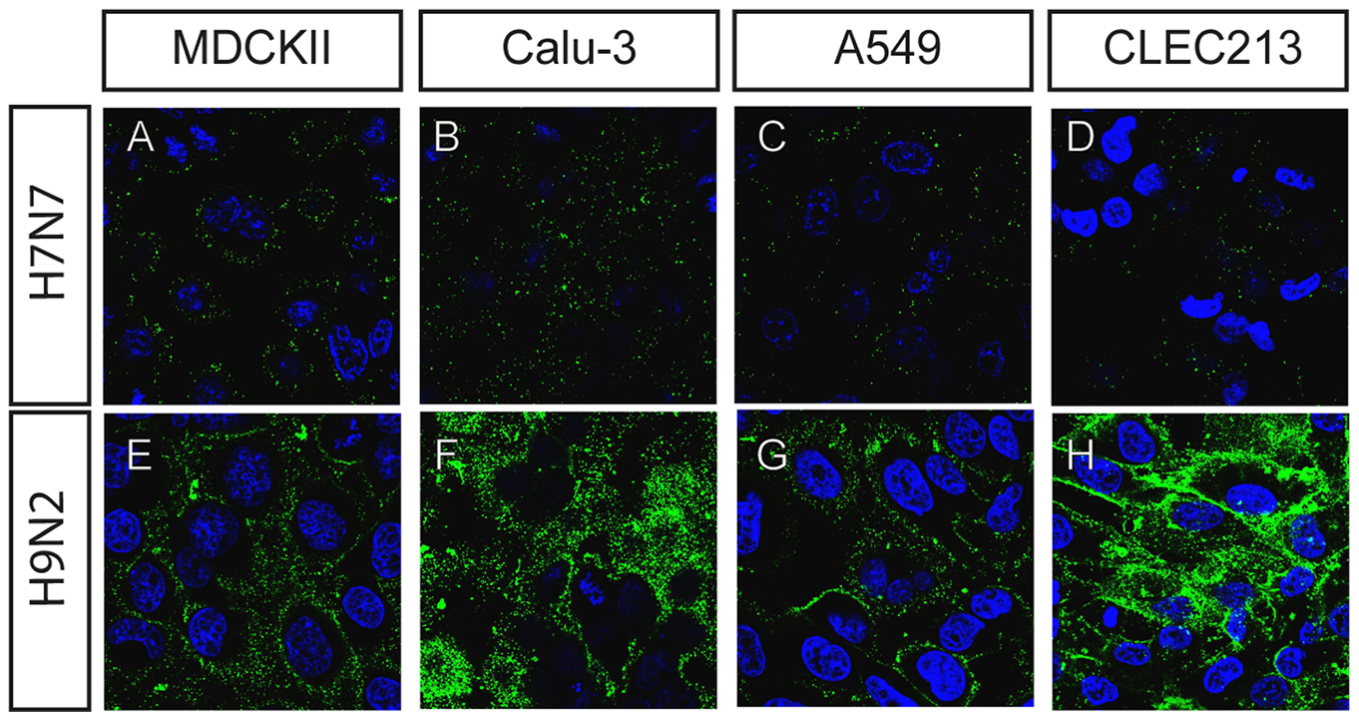
Virus binding tests with different cell lines. Virus (5×10^5^ ffu/ml) was bound at 4°C and stained using specific antibodies against the respective HAs (mouse anti-H7 and rabbit anti-H9, secondary antibodies anti-mouse-FITC and anti-rabbit-FITC respectively). Nuclei were stained with DAPI.

Similar to the results obtained with the soluble HAs, the H9N2 virus bound stronger to all cell lines analyzed than did the H7N7 virus (Fig. 4). Binding of the H9N2 virus to MDCKII, Calu-3, and CLEC123 cells Fig. 4E,F,H) was more efficient than binding to A549 cells (Fig. 4G). These results indicate that the soluble HAs have a similar binding preference as the intact virions and thus are suitable probes to analyze binding sites for influenza viruses.

### Binding to the avian and porcine respiratory epithelium

**Figure 5:**
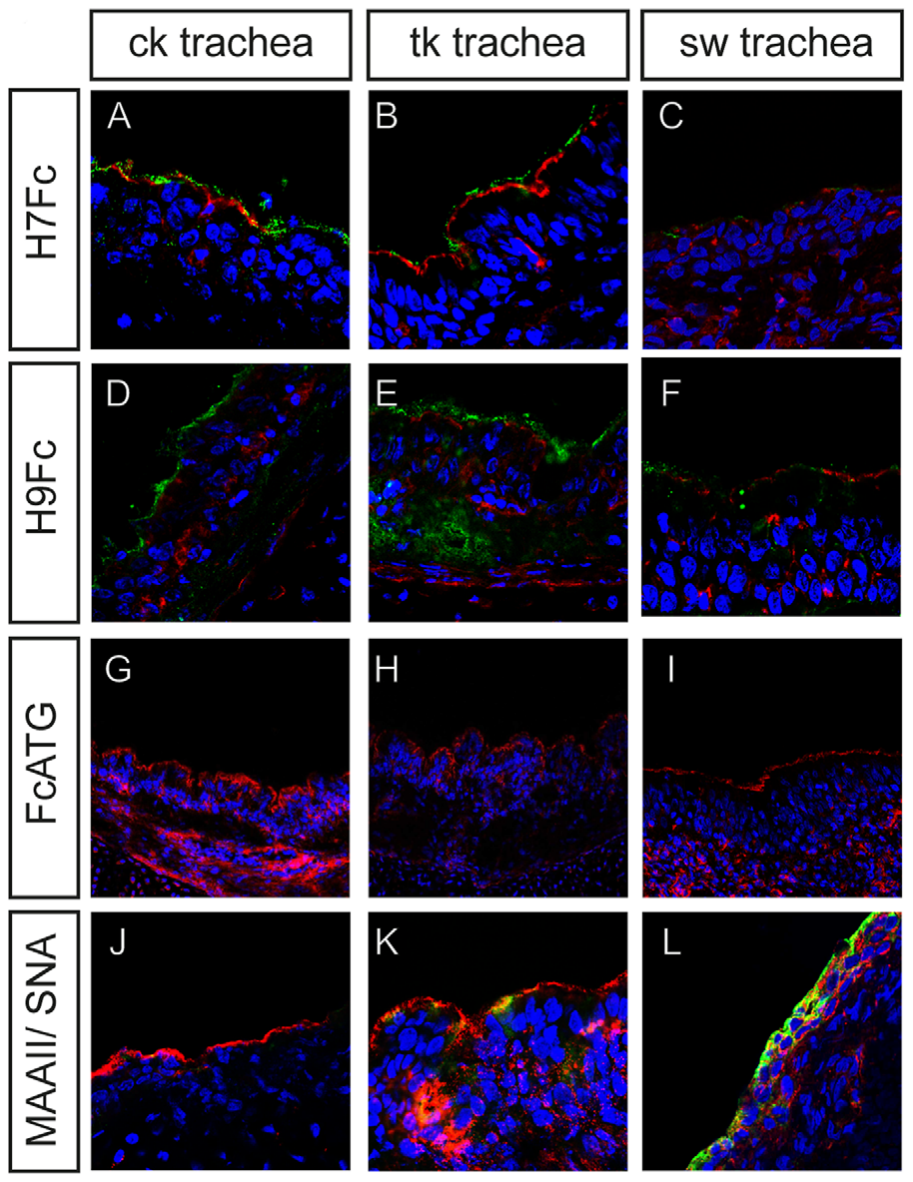
Binding of soluble HAs to tissue sections. Sections of chicken and turkey were incubated with 400 pmol soluble HA. Porcine respiratory epithelium was incubated with 600 pmol soluble HAs to obtain a visible staining. Bound HAs were detected using anti-human IgG-FITC (green). To assess the quality of the epithelium and to visualize cell boundaries, sections were counterstained with phalloidin-rhodamin (red). Nuclei were stained with DAPI. The lower panels show lectin staining of the tissue sections with MAAII-Biotin (red staining by Strepatavidin-Cy3) and FITC-conjugated SNA (green).

To analyze the actual target cells of influenza viruses, we included cryosections of chicken, turkey and swine trachea in our analysis. To visualize single cells and to assess the quality of the sections we performed a counterstaining with phalloidine-rhodamine to stain F-actin (red). The green fluorescence indicates that both H7Fc (Fig. 5A,B) and H9Fc (Fig. 5D,E) bound efficiently to the two avian epithelia lining the tracheal lumen. In the turkey trachea, the binding of soluble H9 HA was also observed in deeper cell layers (Fig. 5E). In the tracheal epithelium of pigs, only very weak fluorescent signals were observed in the sample incubated with H7Fc (Fig. 5C). Binding of H9Fc was more efficient and clearly detectable (Fig. 5F) but the fluorescent intensities were lower than those observed in the avian samples (Fig. 5D,E). In the negative control (FcATG), only the phalloidine staining is detectable demonstrating the specificity of the HA staining (Fig. 5I–L).

Staining by plant lectins was determined by co-staining experiments. On the trachea of chicken and turkey, a clear predominance of MAAII staining (red) was observed (Fig. 5J,K). Among the two avian samples, only the turkey trachea showed some SNA staining (green), which was, however, much less pronounced than the MAAII staining. This result suggests, that the avian trachea expresses predominantly α2,3-linked and only low amounts of α2,6-linked sialic acids which is in agreement with previous lectin staining reports [10], [24]. In the porcine respiratory tract, the opposite staining characteristics were observed (Fig. 5L). The tracheal epithelial cells lining the airway lumen were efficiently stained by SNA (green). MAAII staining was very limited on the surface of the ciliated epithelium but clearly visible in lower cell layers. This is in accordance with other studies that investigated the sialic acid distribution of the porcine respiratory epithelium using lectins [12]–[14].

#### Glycan array analysis

**Figure 6:**
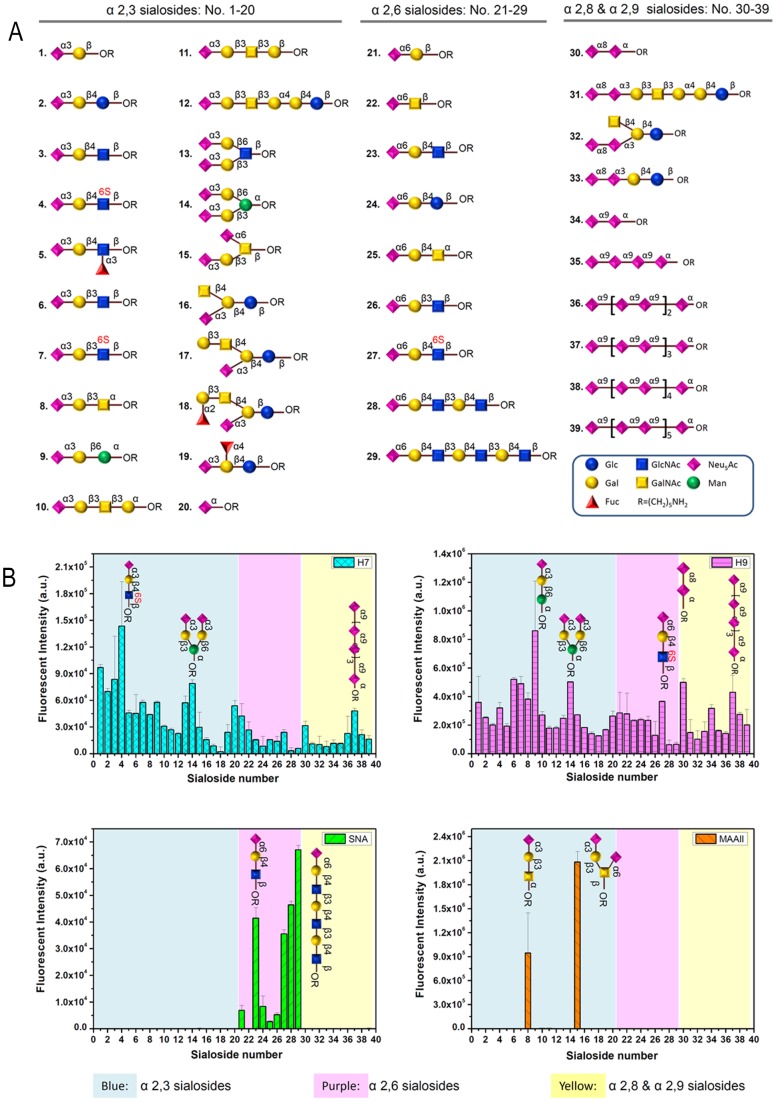
Glycan array analysis of soluble hemagglutinins and lectins. (A) Glycans printed on the array (B) Soluble hemagglutinins (H7Fc and H9Fc) as well as the plant lectins SNA and MAA were subjected to glycan array analysis.

To investigate the binding activities of the soluble HAs and the plant lectins to specific sialoglycoconjugates with α2,3, α2,6, and α2,8/9 linkages, the proteins were subjected to a glycan array analysis. A collection of 39 glycan structures was used comprising oligosaccharides with the following terminal disaccharides: NeuNAc-α2,3-Gal (twenty glycans), NeuNAc-α2,6-Gal (nine glycans), and NeuNAc-α2,8/9-NeuNAc (ten glycans) (Fig. 6A).

Remarkably, of the 39 glycans analyzed, MAAII bound only to two: NeuNAc-α2,3-Gal-β1,3-GalNAc and NeuNAc-α2,3-Gal-β1,3(NeuNAc-α2,6)-GalNAc which is in agreement with data reported by others [25]. SNA detected most efficiently four glycans that had the following terminal structure in common: NeuNAc-α2,6-Gal-β1,4-GlcNAc (Fig. 6B). The soluble hemagglutinin H7Fc showed strongest binding signals with glycans in which the disaccharide Neu5Ac-α2,3-Gal was β1,3-linked to GlcNAc (glycan 4) or Glc (glycan 2). The reaction pattern of H7Fc shows clearly that linkage types and sugar residues next to the terminal disaccharide may have a pronounced effect on the recognition by influenza hemagglutinins. A β1,4-linkage between the terminal disaccharide and GlcNAc (glycans 2–4) was preferred over a β1,3-linkage (glycans 6-8). On the other hand, a β1,3-linkage to mannose was favorable (compare glycans 9,13,14). The modification of the third sugar residue also greatly affected the binding efficiency. A fucose residue α1,3-linked to GlcNAc (glycan 5) was detrimental for the recognition by H7Fc whereas a sulfate group at position C-6 (glycan 4) rather enhanced the interaction. Weak binding was also detected to glycans of the NeuNAc-α2,6-Gal group and the NeuNAc-α2,8/9-NeuNAc group. H9Fc bound to all glycans analyzed though with different efficiency. The overall binding was stronger when compared to H7Fc. The strongest signals were obtained with glycans where the terminal disaccharide NeuNAc-α2,3-Gal was β1,6-linked to Man (glycan 9) or β1,3-linked to GlcNAc (glycan 6). The terminal disaccharide NeuNAc-α2,6-Gal was recognized best when it was attached to the third sugar in a β1,4 (glycans 23–25,27) rather than in a β1,3-linkage (glycan 26). Whether the third sugar was a glucose, an N-acetylglucosamine or an N-acetylgalactosamine (glycans 23–25) was of minor importance but a sulfate group at the N-acetylglucosamine residue (glycan 27) enhanced the interaction with H9Fc. In addition to NeuNAc-α2,3/6-Gal, H9Fc recognized Neu5Ac-α2,8(and2,9)-NeuNAc with high efficiency. Depending on the context of the disialosides, the signals were even higher than those obtained with α2,6-linked oligosaccharides.

Taken together, the glycan array analysis reveals that the H7 and H9 HAs show pronounced differences both in the binding efficiency and in the sialylated glycan structures that are preferentially recognized. Even more striking is the difference between the HAs and the plant lectins. Irrespective of the terminal disaccharide that is preferentially recognized, the plant lectins show a more narrow binding specificity whereas the hemagglutinins recognize a broader spectrum of oligosaccharides. Above all, the glycans bound with highest efficiency by plant lectins are recognized by the influenza hemagglutinins only with low preference. Overall, analysis by plant lectins may provide a misleading picture on glycan structures serving as HA receptors.

## Discussion

Our results show that soluble hemagglutinins may be used to analyze the interaction with sialoglycoconjugates. They have the potential to serve as lectins for identification of sialylated macromolecules. The chimeric nature of this tool with the ectodomain of the hemagglutinin connected to the Fc portion of a human IgG molecule provides a convenient feature for purification of soluble protein and for detection of bound protein. Fc is a dimeric molecule made up from disulfide-linked monomers. Electrophoretic analysis under non-reducing conditions revealed that the preparations of soluble hemagglutinins contained – in addition to dimers also multimeric forms of the chimeric protein (not shown). As the native membrane-bound hemagglutinin is a trimer, HAFc may appear not to be an optimal form for a soluble functional hemagglutinin. Therefore, we also attached the HA ectodomain to the trimerization domain of a leucine zipper protein. However, this chimeric protein was not more efficient in its binding properties when compared to Fc constructs (data not shown). Because of the advantages of the Fc tag, we preferred to work with the HAFc proteins. The Fc tag is not only convenient for purifying the proteins but also for comparing different hemagglutinins. The same anti IgG antibody was used to detect H7Fc and H9Fc. In this way we could show that binding of H9 to cells is more efficient than binding of H7. For detection of chimeric proteins containing a trimerization domain, one would have to use an anti-H9 and an anti-H7 antibody. In this case, a comparative binding analysis would have to take into account different binding affinities of the two antibodies. Therefore, the Fc-tagged chimeric HA constructs are a more suitable tool.

Our analysis demonstrates that the two influenza hemagglutinins have a broader binding specificity than have the two plant lectins. Both HAs recognized oligosaccharides terminating with the disaccharides NeuNAc-α2,3-Gal, NeuNAc-α2,6-Gal, or NeuNAc-α2,8/9-NeuNAc. In general, H9Fc bound to the glycans with higher affinity than did the H7Fc protein. There were pronounced differences in the type of oligosaccharide(s) that was/were preferentially recognized. The glycan array analysis clearly shows that sugars and linkage types next to the disialoside structure may also be crucial components of the recognition motif. Even a modification of the third sugar by a sulfate group or a fucose residue may be of critical importance for optimal binding of hemagglutinins to sialooligosaccharides. Therefore, two plant lectins that are used to differentiate between α2,3 and α2,6-linked sialic acids are not reliable indicators of the presence of binding sites for influenza viruses.

Interestingly, the hemagglutinins also recognize NeuNAc-α2,8/9-NeuNAc structures. Binding to α2,8-linked sialic acids has been shown for other influenza viruses [35], [36]. This linkage type is underestimated in the evaluation of the receptor binding activities of influenza viruses, because the respective disialosides are not substrates for the plant lectins MAA and SNA. It would be interesting to know whether site-directed mutagenesis of the HA can increase this binding specificity with a concomitant loss of the recognition of other linkage types. This would generate a valuable tool in the analysis of sialic acids.

Some H9 HAs are known to differ from the HA proteins of other avian influenza A viruses by having an increased capability to recognize α2,6-linked sialic acids [6]. Growth in certain hosts like quails and turkeys may result in a point mutation that further enhances the binding to oligosaccharides terminating with NeuNAc-α2,6-Gal as has been shown by glycan array analysis [22]. The H9Fc used in this study does not contain this mutation at the receptor binding site; otherwise, it would differ even more in its binding activity from that of H7Fc.

Though both HAs are derived from chicken viruses, they differ in the receptor binding profile in glycan array analysis. These differences are reflected in the binding tests of cultured cells and tissue samples indicating their relevance for the distribution of the influenza virus receptors. In this context, it is interesting that the differential binding of the H7Fc is also reflected in the replication of the viruses. Consistent with the efficient binding of both H7Fc and H9Fc, H9N2 and H7N7 influenza viruses grow to similar titers in tracheal organ cultures. On the other hand, the more efficient binding of H9Fc and H9N2 virus to Calu-3 cells compared to H7Fc and H7N7 virus affects also the replication as indicated by an about 100-fold higher amount of infectious H9N2 virus released from these cells (Erdt, Bohm, Herrler, unpublished). Though the number of binding sites for H7 viruses are low on Calu-3 cells, they appear to be even lower in A549 cells. Despite the presence of MAAII staining on both A459 cells and Calu-3 cells in approximately equal intensities H7Fc binding could only be observed on Calu-3 cells. A459 appear to express no or only low levels of suitable H7 receptors. However, they contain glycans recognized by H9Fc. Gambaryan et al 2012 [26] have shown that most H7 HAs preferentially bind to sulfated receptor determinants that were also recognized by H7Fc as shown by our glycan array analysis. Binding tests in the porcine trachea showed that this tissue contains binding sites for both H9Fc and H7Fc, although the latter exhibited much less binding. Both virus subtypes are able to replicate in differentiated respiratory epithelial cells [14] and H9N2 viruses circulate in the pig population in Southeast Asia [27], [28].

The ability to bind and to replicate in the respiratory tissues of poultry species and pigs shows the pandemic potential of these viruses and indeed H7 and H9 viruses are known to readily infect humans [29]–[31]. The recent isolation of H7N9 influenza virus from humans demonstrates this risk. Interestingly, the internal genes of this virus are derived from H9N2 viruses whereas the surface glycoproteins are derived from different subtypes. The human H7N9 virus has an increased ability to recognize α2,6-linked sialic acids compared to avian H7 viruses [32], [33]. However, it still recognizes both α2,3- and α2,6-linked sialic acids similar to the H9 hemagglutinin used in our study. By constrast, some H9 influenza viruses have adapted to preferentially bind to glycans containing α2,6-linked sialic acids [5], [6], [34]. Therefore, the mutations in the hemaglgutinins of the H7N9 viruses isolated from humans may explain the potential of the viruses to grow in human cells but not the increased pathogenicity for human patients compared to H9N2 viruses. Mutations in other genes, e.g. in PB2, may also contribute to the pathogenicity of the human H7N9 viruses. These findings show that the sialic acid binding activity is an important but not the only determinant for the zoonotic potential of influenza viruses. They also show, that in the future it is important to analyze not only the preferred receptor determinants for influenza viruses but to identify the actual receptor molecules on the surface of the target cells. For this work, Fc-coupled hemagglutinins should be a valuable tool.

The information provided by the glycan array analysis shows which sialooligosaccharides are preferred receptor determinants for influenza hemagglutinins but not how these receptors are distributed on cells and tissues. To further elucidate the role of sialic acid in virus entry, it is necessary to know how many surface glycoproteins containing the recognition motif are required to enable influenza viruses to enter the target cell. Further questions to be addressed are: Can surface components that contain receptor determinants which are recognized with lower affinity serve as virus receptors, too, and if so, how many receptor molecules are required? How important is it whether the receptor determinant is present on a glycoprotein or on a glycolipid?

Lectin binding gives a first hint whether sialic acids suitable for virus attachment are present on the cell surface. However, in this respect, soluble hemagglutinins are more reliable tools than plant lectins. This conclusion is based on our binding tests with cells and tracheal tissues from different species. Binding of soluble hemagglutinins went in parallel with binding of intact virions of the same subtype, and was a better match than the binding of the plant lectins. This is not surprising when the narrow specificity of the plant lectins is taken into account. So far, there are only few reports about the application of soluble HAs to analyze the binding activity of influenza A viruses [35]–[37]. Soluble hemagglutinins may be valuable tools in the future to analyze the interaction of influenza viruses with target cells in more detail.

## Materials and Methods

### Ethics statement

For chicken and turkey, a study approval from an ethics committee was not required as working with avian embryos is not regulated by the German Animal Welfare Act (http://www.bmelv.de/SharedDocs/Rechtsgrundlagen/T/Tierschutzgesetz.html), as confirmed by the animal welfare official of the University of Veterinary Medicine Hannover. The Clinic for Poultry has a hatching facility to incubate and hatch eggs of different poultry species. Furthermore, animal facilities are available at the University of Veterinary Medicine Hannover, to house different animal species including poultry. Embryos used for preparation of trachea sections and chicken kidney cells were humanely sacrificed by decapitation and did not undergo any procedures prior to this. The personnel involved in this procedure had been educated to sacrifice the embryos in a humane and quick way. Virus was propagated in 10 d old embryonated chicken eggs, maintained for three days and subsequently chilled at 4°C for 24 h.

Pigs used for the preparation of trachea sections were kept in the Clinic for Swine and Small Ruminants, University of Veterinary Medicine Hannover, for demonstration and students' veterinary training (approval number 33.9–42502–05–09A627). All studies were carried out in strict accordance with the recommendations of the European Convention for the Protection of Vertebrate Animals used for Experimental and Other Scientific Purposes (European Treaty Series, nos. 123 [http://conventions.coe.int/Treaty/en/ Treaties/Html/123.htm] and 170 [http://conventions.coe.int/ Treaty/en/Treaties/Html/170.htm]. The protocol was approved by the national permitting authorities (animal welfare officer of the University of Veterinary Medicine, Lower Saxony State Office for Consumer Protection and Food Safety). All measures were in accordance with the requirements of the national animal welfare law. Killing and tissue sampling were performed under sodium pentobarbital anesthesia, and all efforts were made to minimize suffering.

### cDNA, viruses, cell lines and tissues

The cDNA for the influenza hemagglutinin of A/chicken/Netherland/621557/2003 (H7N7) HPAI was obtained from the index farm of the 2003 Dutch avian influenza virus outbreak. Full-length cDNA of the HA genome segment was cloned in transcription vector pPolSapRib [15], [16]. The cDNA of the H9 hemagglutinin was derived from A/chicken/Emirates/R66/2002 (H9N2) [17], [18]. The latter strain was also used for virus binding. The H7N7 virus used for virus binding studies was the low-pathogenic strain A/duck/Potsdam/15/80 (H7N7) LPAI.

MDCKII and HEK 293T cells were cultivated in Dulbecco's modified Eagle's medium (DMEM; Gibco) with 10% fetal calf serum (FCS; Biochrom), Calu-3 cells in Eagle's minimal essential medium (EMEM; Gibco) and 5% FCS, 1% non-essential amino acids (PAA) and 1% sodium pyruvate (PAA). A459 cell were kept in Ham's F12 medium (PAA) including 10% FCS. Chicken lung epithelial CLEC213 cells have been described recently [19].

Turkey and chicken trachea were obtained from 25 and 20 days old SPF chicken embryos, cut into small rings and mounted in freezing medium. Cryosections, 10 µm thick, were generated for analysis by fluorescence microscopy.

Primary chicken kidney cells (PCKC) were prepared from the kidneys of chicken embryos and seeded on coverslips as described previously [20].

Porcine trachea was obtained from three months old crossbred pigs housed in the Clinics for Swine and Small Ruminants and Forensic Medicine at the University of Veterinary Medicine, Hannover. The trachea was cut into smaller rings and each ring into 4 pieces that were mounted in freezing medium and used for cryosectioning.

### Molecular cloning and preparation of soluble hemagglutinins

To obtain soluble hemagglutinins, the cytoplasmic tail and transmembrane anchor were deleted using PCR. Then the HA ectodomains of H7 (Genbank accession AY338458: nucleotides 1–1.574 corresponding to amino acid positions 1–525) and of H9 (Genbank accession CY076723: nucleotides 1–1.555 corresponding to amino acid positions 1–520) were cloned into the pCG1-Fc vector containing a human IgG Fc domain (kindly provided by Jörg Glende). This vector is derived from the pCG1 plasmid (R. Cattaneo, Mayo Clinic College of Medicine, Rochester, Minnesota, USA).

The plasmids coding for the Fc-tagged HA ectodomains were used for transfection of HEK 293T by the calcium phosphate precipitation method. Medium was changed after 16 h and supernatants were collected after 72 h and up to 96 h post-transfection. Then, the supernatants were purified by FPLC using the HiTrap™ Protein A HP Columns (GE Healthcare). The proteins were eluted from the columns with 0.1 M sodium citrate, pH 3.0, into 1 M Tris, pH 9, as neutralization buffer.

Protein concentration was measured at 280 nm in a photometer and calculated according to Lambert Beer Law. Protein quality and purity were analyzed as follows. After separation via SDS polyacrylamide gel electrophoeresis, soluble proteins were either transferred to nitrocellulose membrane by a semi-dry Western blot procedure [21] or SDS gels were directly Coomassie stained. After blocking non-specific binding sites of the membrane for 1 h, soluble HAs were detected with horse radish peroxidase conjugated anti-human IgG PO (1:10.000, Sigma Aldrich). Aliquots of the purified proteins were stored undiluted at −20°C.

### Binding tests

Permanent cell lines were grown to confluency on coverslips and fixed with 3% paraformaldehyde. For the binding test, 100 pmol of protein diluted in 1% BSA were applied per coverslip for 1 h at 4°C. The HAs were detected using an anti-human-FITC antibody (Sigma Aldrich) for 45 min at RT. The coverslips were mounted in mowiol containing DAPI to stain the nuclei.

For binding tests on cryosections of the respiratory epithelium, different concentrations of soluble HAs ranging from 400 pmol to 600 pmol were used. Detection and mounting was similar to the coverslip procedure.

For detection of sialic acids, the lectins *Maackia amurensis* agglutinin II (MAAII, dilution 1∶200, Vectorlabs) and FITC-conjugated *Sambucus nigra* agglutinin (SNA-FITC, dilution 1∶100, Vectorlabs) were used. MAAII was biotin-conjugated and detected by FITC- or Cy3- conjugated streptavidin. Fluorescent staining was visualized with a confocal scanning microscope (Leica TCS SP5 AOBS). Quantification was performed using Image J software by measuring overall green fluorescence of microscopic pictures. The values obtained in this way, were used – after correction for the control sample – to determine the ratio between two samples (comparing either two hemagglutinins or two cell types) and thus the relative fluorescence values that are mentioned in the result section.

To remove sialic acids from the cell surface, cells were treated with 200 mU Type V neuraminidase from *Clostridium perfringens* (Sigma Aldrich) for 1 h at 37°C prior to fixation.

For binding tests with intact virions we used 20 µl of a virus suspension, 5×10^5^ ffu/ml, and incubated the coverslips as described above for the soluble hemagglutinins. The H9N2 virus was detected with a polyclonal rabbit anti-H9N2 serum. The H7 HA was detected by a mouse monoclonal antibody. Both antibodies were kindly provided by Wolfgang Garten, Philipps-Universität Marburg. The respective secondary antibodies were FITC-conjugated (1∶500, Sigma Aldrich).

### Glycan array fabrication and HA binding

The generation of the glycan array has been described recently [14], [22].

All experiments were repeated three times. Soluble hemagglutinins (HAFc) were overlaid onto the arrays and incubated at room temperature for 1 hour. Slides were subsequently washed by successive rinses in PBS-0.05% Tween, PBS, and deionized water three times. Bound viruses were labeled by primary antibodies. The slides were gently rocked at room temperature for another 1 hour. After the different washing steps, binding was detected by overlay with dye-labeled secondary antibodies. The slides were air-dried and scanned with a microarray fluorescence chip reader (GenePix 4300B, Molecular Devices). The PMT gain was set to 450. The resulting images were analyzed with GenePix Pro 7 (Molecular Devices) to locate and quantify the fluorescence intensity of all of the spots on the grid.
